# Efficacy of Ravulizumab During the Acute Phase of Neuromyelitis Optica Spectrum Disorder

**DOI:** 10.1155/crnm/1518364

**Published:** 2026-04-06

**Authors:** Koji Tsuzaki, Goro Mitsui, Yasunobu Inagaki, Naoko Uehara, Shinichi Wada, Toshiaki Hamano

**Affiliations:** ^1^ Department of Neurology, Kansai Electric Power Hospital, 2-1-7 Fukushima Fukushima-ku, Osaka, 553-0003, Japan, kanden-hsp.jp; ^2^ Division of Clinical Neurology, Kansai Electric Power Medical Research Institute, 2-1-7 Fukushima Fukushima-ku, Osaka, 553-0003, Japan, kepmri.org; ^3^ Division of Sleep Medicine, Kansai Electric Power Medical Research Institute, 2-1-7 Fukushima Fukushima-ku, Osaka, 553-0003, Japan, kepmri.org

## Abstract

**Background:**

Ravulizumab is effective in preventing relapse of neuromyelitis optica spectrum disorder (NMOSD). However, its efficacy during the acute phase of NMOSD has rarely been reported.

**Case:**

We report the case of a 66‐year‐old woman with anti‐aquaporin‐4 antibody‐positive NMOSD. Six years ago, the patient had impaired vision and visual field loss in her right eye and received steroid therapy at another hospital for 6 months, after which no further treatment was given. On admission, she presented with weakness in both lower limbs, numbness from the chest to the lower body, and impaired bladder sensation. Neurological examination revealed bilateral lower limb weakness and hypoesthesia below the 4th thoracic spinal segment. The Expanded Disability Status Scale (EDSS) score was 3.5. Cerebrospinal fluid examination revealed mild cytosis, elevated protein levels, an elevated immunoglobulin G index, and positive oligoclonal bands. Magnetic resonance imaging revealed longitudinally extensive transverse myelitis from the 2nd to 8th thoracic vertebral levels. She was diagnosed with a relapse of NMOSD. After one course of steroid pulse therapy, she developed complete paraplegia in both legs. The EDSS score was 8.5. Immunoadsorption therapy, prednisolone, and azathioprine were initiated; however, these treatments were ineffective. Ravulizumab was administered immediately after immunoadsorption therapies. Treatment with prednisolone, azathioprine, and ravulizumab was continued. Her symptoms gradually improved, and prednisolone was gradually tapered and discontinued. She could walk independently after 6 months. The EDSS score was 3.5 at the time of discharge.

**Conclusion:**

Ravulizumab may be a viable treatment option when administered during the acute phase of NMOSD.

## 1. Introduction

Neuromyelitis optica spectrum disorder (NMOSD) is an inflammatory disease of the central nervous system (CNS) that predominantly affects the optic nerves and spinal cord and less, frequently, the brainstem [1]. In many cases, NMOSD is associated with the presence of pathogenic immunoglobulin G (IgG) antibodies against aquaporin‐4 (AQP4), the most common water channel protein in the CNS [1]. Five preventive immunotherapeutic drugs have recently been approved for treating AQP4‐positive NMOSD in Japan as follows: eculizumab, ravulizumab, inebilizumab, rituximab, and satralizumab. Eculizumab is a humanized monoclonal antibody that binds to complement protein C5 and disrupts the terminal complement cascade [2]. Ravulizumab is also a monoclonal antibody that targets complement factor C5 but has a half‐life that is four times longer than that of eculizumab [2]. Eculizumab and ravulizumab are considered effective not only in preventing relapse but also in improving symptoms during the acute phase of NMOSD. We report the case of a patient with NMOSD who achieved a favorable outcome following ravulizumab administration immediately after steroid pulse and immunoadsorption therapies.

## 2. Case Presentation

The patient was a 66‐year‐old Japanese woman who had experienced impaired vision (from 0.7 to 0.01) and superior altitudinal field loss in the right eye 6 years before presentation. She had been diagnosed with NMOSD with positive AQP4 antibodies at another hospital. After three courses of steroid pulse therapy, oral prednisolone was initiated. However, it was discontinued within 6 months, and no further treatment was administered. Three days before admission, the patient experienced a dull pain in the back and mild nausea. On the day of admission, the patient had weakness in both lower limbs, numbness from the chest to the lower body, and difficulty feeling the urge to urinate. She had no bowel movement for 3 days. Her comorbidities included hypercholesterolemia, and she was taking atorvastatin.

Neurological examination revealed weakness in both lower limbs (Medical Research Council [MRC] Scale: iliopsoas 4/4, quadriceps 5/5, knee flexors 4/4, tibialis anterior 4/4, and gastrocnemius 5/5). She experienced tactile, thermal, and vibrational hypoesthesia below the 4th thoracic spinal segment. She had difficulty perceiving the urge to urinate but rarely experienced urinary incontinence. Visual acuity was 0.6 and 0.9 on the right and left, respectively, with no visual field defects. The deep tendon reflexes were within the normal range. Pathological reflexes were not observed. In functional systems (FS), the pyramidal score was 3, sensory was 2, bowel/bladder was 2, and visual was 2; other scores were 0. The Expanded Disability Status Scale (EDSS) score was 3.5. Magnetic resonance imaging revealed a long lesion showing high‐intensity signals on T2‐weighted images within the spinal cord from the 2nd to 8th thoracic vertebral levels, with mild swelling and contrast enhancement. No abnormal signals were observed in the optic nerves, cerebral hemispheres, or the medulla oblongata. Cerebrospinal fluid examination revealed mild cytosis (cell counts: 36/μL, mononuclear: 22/μL, and segmented nuclei: 14/μL), elevated protein levels (82.5 mg/dL), an elevated IgG index (0.84), and positive oligoclonal bands. Blood tests showed positive anti‐AQP4 antibodies (> 40 nmol/L, enzyme‐linked immunosorbent assay) and no findings suggestive of other myelitis (herpes simplex virus, varicella zoster virus, human immunodeficiency virus, syphilis, systemic lupus erythematosus, antineutrophil cytoplasmic antibody‐associated vasculitis, or Sjögren’s syndrome), paraneoplastic neurological syndrome, or malignant lymphoma. The patient was diagnosed with a relapse of NMOSD.

**FIGURE 1:**
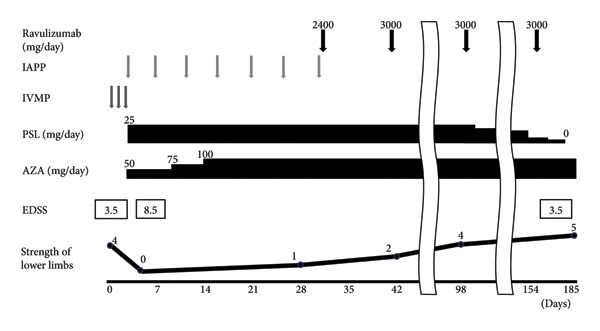
Clinical course and treatment. IAPP, immunoadsorption plasmapheresis; IVMP, intravenous methylprednisolone; PSL, prednisolone; AZA, azathioprine; EDSS, Expanded Disability Status Scale; lower limb muscle strength, expressed as the Medical Research Council Scale.

Although steroid pulse therapy (methylprednisolone, 1 g/day) was administered from the day of admission (the fourth day after the symptoms appeared), her symptoms progressed daily, resulting in complete paraplegia and loss of sensation in lower extremities (EDSS score, 8.5). Rehabilitation was initiated at the time of admission and continued throughout the hospitalization. Immunoadsorption therapy was subsequently initiated two times a week for seven sessions. Oral administration of prednisolone 25 mg (0.5 mg/kg) and azathioprine 100 mg (2 mg/kg) was also initiated. On day 18 of hospitalization, the patient’s sensory impairment had improved slightly. On day 28, muscle contractions of the lower limbs were observed. The patient’s urinary retention also improved. However, the treatment showed limited efficacy, and no further improvements were expected. Two weeks after the first meningococcal ACWY vaccination, 2400 mg of ravulizumab was administered on day 28 of hospitalization. Prophylactic administration of third‐generation cephalosporin antibiotics was not performed. The genetic variant c2654G ⟶  A was not identified. On day 28 (just before first ravulizumab administration), 50% hemolytic complement (CH50) assessed using the modified Mayer’s method was 47.1 U/mL and it decreased to 23.4 U/mL on 42 day (14 days after first ravulizumab administration). On day 42 of hospitalization, the MRC scale for the lower limbs was 2, and ravulizumab (3000 mg) was administered. Thereafter, ravulizumab administration was repeated every 8 weeks. After the initiation of treatment with ravulizumab, her muscle strength gradually improved. On day 99, the MRC Scales were iliopsoas 4/3, quadriceps 5/5, knee flexors 3/3, tibialis anterior 5/5, and gastrocnemius 5/5. The second meningococcal vaccination was administered on day 99. Prednisolone was gradually tapered and discontinued on day 171. CH50 on day 171 was 17.3 U/mL (15 days after the fourth administration of ravulizumab). Although the patient had mild muscle weakness in both legs and numbness in the lower body, she could walk independently when discharged on day 185. The MRC scale for the lower limbs was almost 5. In FS, the pyramidal score was 3, sensory was 2, bowel/bladder was 0, and visual was 2. The EDSS score was 3.5 at the time of discharge (Figure 1). Since discharge, treatment with ravulizumab and azathioprine has been continued, and the patient has had no recurrence. So far, there had been no side effects from ravulizumab.

## 3. Discussion

Herein, we report a case where symptoms improved following ravulizumab administration immediately after steroid pulse and immunoadsorption therapies during the acute phase of NMOSD.

Eculizumab demonstrated significant efficacy in preventing relapse of NMOSD, particularly in patients with positive AQP4 antibodies. In the phase 3 trial (PREVENT trial), adjudicated relapses occurred in only 3% of patients in the eculizumab group and 43% in the placebo group (hazard ratio, 0.06; *p* < 0.001) [3]. In the phase 3 CHAMPION‐NMOSD trial, none of the 58 patients with NMOSD in the ravulizumab group experienced relapse during 84.0 patient‐years of treatment. However, 20 patients showed adjudicated relapses in the placebo group of the PREVENT trial during 46.9 patient‐years [4]. Ravulizumab’s longer half‐life allows dosing every 8 weeks, reducing infusion burden compared to eculizumab.

To assess the pharmacokinetics and pharmacodynamics of ravulizumab in a phase 3 trial (CHAMPION–NMOSD), baseline and trough analyses were performed 90 min before infusion, and peak post‐dose samples were collected 60 min after infusion [5]. Immediate and complete terminal complement inhibition (free C5 serum concentrations < 0.5 μg/mL) was observed at the end of the first ravulizumab infusion and was sustained throughout the primary treatment period [5]. Therefore, the therapeutic effects of ravulizumab via C5 inhibitory action apparently appear immediately after infusion, and ravulizumab may also be a viable treatment when administered during the acute phase of NMOSD.

Only one previous study has demonstrated the efficacy of ravulizumab during this phase of NMOSD [6]. In this case, the symptoms worsened despite steroid pulse therapy and plasma exchange (PE). After ravulizumab was initiated, symptom deterioration ceased and the EDSS score improved by 2 points [6].

A few case reports have described the efficacy of eculizumab during the acute phase of NMOSD [7–12]. Eleven cases have been reported; in all cases, steroid pulse therapy and PE were performed before eculizumab administration. Additionally, the EDSS or visual acuity improved after eculizumab administration (Table 1). These results suggest that eculizumab is a viable treatment option for reducing the symptoms of severe NMOSD attacks, although steroid pulse therapy or PE may have exhibited delayed effects.

**TABLE 1:** Reports of eculizumab and ravulizumab during the acute phase of neuromyelitis optica spectrum disorder.

Drug	Reference	Sex	Age	Treatment before ECU/RVZ	Days from the onset of attack to ECU/RVZ	EDSS		Visual acuities (Rt/Lt)	
						Before ECU/RVZ	After ECU/RVZ	Before ECU/RVZ	After ECU/RVZ
ECU	Chatterton et al. [7]	F	46	IVMP, PE	12	NA	NA	0.1/light perception only	NA/CF
	Kaneko et al. [8]	F	51	IVMP, PE	22	NA	2.5	NA/NA	NA/NA
		F	51	IVMP, PE	21	NA	NA	0.4/0.15	0.5/0.4
	Gorriz et al. [9]	F	47	IVMP, PE	NA	9.5	8.5	NA/NA	NA/NA
	Enriquez et al. [10]	M	12	IVMP, PE	NA	9.5	NA	NA/NA	NA/NA
	Watanabe et al. [11]	F	50	IVMP, PE	46	6.5	4.5	NA/NA	NA/NA
		F	53	IVMP, PE	41	4.0	3.5	1.2/1.2	1.2/1.2
		F	78	IVMP, PE	30	7.5	7.5	0.06/0.05	0.4/0.3
		F	54	IVMP, PE, IVIg	42	NA	NA	0.08/1.2	0.1/1.2
		M	93	IVMP, PE	61	6.5	6.5	CF (20 cm)/0.15	0.01/0.2
	Soni et al. [12]	F	10	IVMP, PE	16	NA	NA	NA/NA	NA/NA

RVZ	Valdés et al. [6]	F	58	IVMP, PE	12	9.0	7.0	NA/NA	NA/NA
	Our case	F	66	IVMP, PE	28	8.5	2.0	0.6/0.9	NA/NA

*Note:* ECU, eculizumab; RVZ, ravulizumab; IVMP, intravenous methylprednisolone; IVIg, intravenous immunoglobulin.

Abbreviations: CF, counting fingers; EDSS, Expanded Disability Status Scale; NA, not available; PE, plasma exchange.

Steroid pulse and immunoadsorption therapies were performed before ravulizumab administration in our case. Magana et al. reported that patients with central inflammatory demyelinating diseases who responded to PE improved within a median of 4 days (range: 1–100 days). Conversely, 6% of responders had a delayed response, 60–100 days after PE treatment [13]. Therefore, the marked improvement in symptoms of MNOSD in our case might not be the effect of ravulizumab but may reflect the delayed effects of steroid pulse therapy or PE. It is not possible to avoid steroid pulse therapy or PE and use only ravulizumab during the acute phase of NMOSD. Large‐sample‐size studies are needed to determine whether adding ravulizumab to steroid pulse therapy and PE improves prognosis. Rehabilitation may also have contributed to improving muscle strength.

Amano reported that the value of CH50 measured immediately after administration fell below the detection limit [14]. However, in our case, CH50 was measured approximately two weeks after ravulizumab administration. The difference in the timing of CH50 measurement is considered the reason why CH50 did not fall below the detection limit in our case.

Although ravulizumab increases the risk of developing infections such as meningococcal infections [4], no infections have occurred in our cases so far.

In conclusion, treatment with ravulizumab during the acute phase of NMOSD may be useful. Further research is necessary to validate our findings.

## Author Contributions

Conceptualization: Koji Tsuzaki; investigation: Koji Tsuzaki, Goro Mitsui, and Yasunobu Inagaki; writing–original draft preparation: Koji Tsuzaki; writing–review and editing: Koji Tsuzaki, Goro Mitsui, Yasunobu Inagaki, Naoko Uehara, Shinichi Wada, and Toshiaki Hamano; supervision: Toshiaki Hamano.

## Funding

The study did not receive any funding.

## Ethics Statement

Ethics Committee of Kansai Electric Power Hospital issued approval #25‐063.

## Consent

Consent for treatment and publication was obtained from all participants in this study.

## Conflicts of Interest

The authors declare no conflicts of interest.

## Data Availability

The data that support the findings of this study are available on request to the corresponding author. The data are not publicly available due to privacy or ethical restrictions.
